# Thoracic Epidural Anesthesia for Modified Radical Mastectomy in a High-Risk Patient: A Case Report With Literature Review

**DOI:** 10.7759/cureus.15822

**Published:** 2021-06-22

**Authors:** Aarti Srivastava, Shaista Jamil, Ankur Khandelwal, Manish Raj, Shalley Singh

**Affiliations:** 1 Anaesthesiology and Critical Care, Sharda University School of Medical Sciences and Research, Greater Noida, IND

**Keywords:** analgesia, breast cancer, general anesthesia, modified radical mastectomy, thoracic epidural anesthesia

## Abstract

Surgery is one of the mainstays of treatment in breast cancers. Typically, modified radical mastectomy (MRM) is done under general anesthesia (GA). However, GA is not a reasonable choice in patients with multiple comorbidities and difficult airways. Thoracic epidural anesthesia (TEA) is a reasonable and safe alternative to GA as it involves blunting of stress response and avoidance of airway handling apart from hemodynamic stability, lower analgesic consumption, superior postoperative analgesia, reduced postoperative nausea and vomiting, earlier resumption of feeding, and shorter duration of hospitalization. We report a case of advanced breast cancer in a 57-year-old female with a co-existing difficult airway, bronchial asthma, and hypertension in whom MRM was conducted successfully under TEA. We also present a comprehensive review of literature on the use of TEA for MRM.

## Introduction

Breast cancer is the most common cancer among Indian females with an age-adjusted rate as high as 25.8 per 100,000 women and mortality of 12.7 per 100,000 women [1]. It can occur at any age but the incidence rates in India begin to rise in the early thirties and peak at ages 50-64 years [2]. Surgery is one of the mainstays of treatment. Modified radical mastectomy (MRM) typically involves removal of the entire breast, including the skin, areola, nipple, and most axillary lymph nodes, but the pectoralis major muscle is spared. MRM is usually done under general anesthesia (GA). We report a case of advanced breast cancer in a 57-year-old female with a co-existing difficult airway, bronchial asthma, and hypertension in whom MRM was conducted successfully under thoracic epidural anesthesia (TEA). We also present a comprehensive review of literature on the use of TEA for MRM. Written informed consent was obtained from the patient for reporting this case for publication.

## Case presentation

A 57-year-old female with left-sided advanced breast cancer was posted for MRM. She was a known case of bronchial asthma since childhood and hypertension for three years. Her usual medications included salbutamol puffs (as when required) for asthma and oral amlodipine (5 mg once daily) for hypertension. However, she was poorly compliant with amlodipine. She had an episode of acute exacerbation of asthma seven days prior to surgery for which she was managed with salbutamol nebulization and IV methylprednisolone. She underwent hysterectomy 18 years back under spinal anesthesia, which was uneventful. There was no history of any allergy to food or drug.

On the evening prior to surgery, her heart rate (HR), blood pressure (BP), and respiratory rate (RR) were 88 beats/min, 140/86 mmHg, and 20/min, respectively. Her investigations were within normal limits except electrocardiogram and chest radiograph which were suggestive of left ventricular hypertrophy. Her airway examination revealed Mallampati Class IV. Due to reactive airway disease, hypertension, and difficult airway, she was counselled for TEA and written consent was taken. She received her usual dose of amlodipine on the morning of surgery.

**Figure 1:**
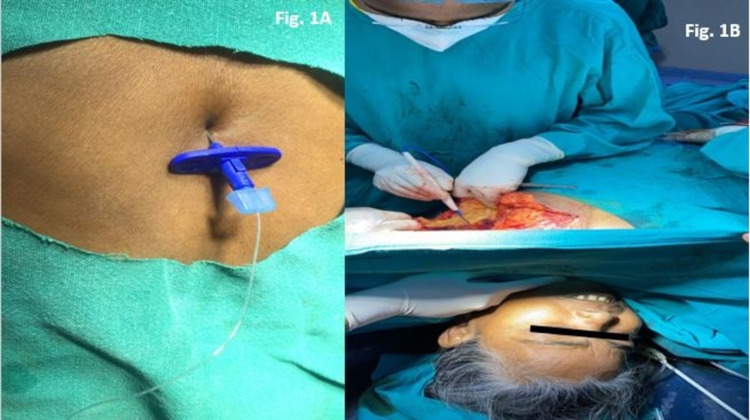
A: Image showing placement of thoracic epidural catheter. B: Image showing calm, comfortable, and awake patient during modified radical mastectomy.

In the operating room, a multi-parameter monitor was attached to the patient. Baseline values were as follows: HR-84/min, BP-130/80 mmHg, and SpO2: 98%. A preloading of 500 ml of Ringer's lactate was done. Under full aseptic and antiseptic conditions, a midline thoracic epidural was performed in a sitting position at the T6/T7 interspinous space using an 18G Tuohy epidural needle. The targeted space was identified by loss of resistance to air. The skin to epidural depth was 6 cm. Around 5 cm of the epidural catheter was inserted in the epidural space in the cephalad direction and the catheter was adhered to the back with adhesive dressing (Figure 1A). After a test dose of 3 ml of 2% lidocaine with adrenaline (1:200000), a loading dose of 10 ml of 0.5% bupivacaine was given slowly in 5 ml aliquots at five-minute intervals. Oxygen was administered via a face mask at 5 litres/min throughout the surgery. A sensory block height of T1 to T7 was achieved bilaterally (assessed by pinprick) which allowed the surgery to commence. The chemo port was inserted through the right subclavian vein. The left breast tissues and tumour were excised en bloc, including fibro-fatty tissues of the left axilla. Towards the end of the surgery, she complained of mild pain which was effectively managed with an additional 5 ml of 0.5% epidural bupivacaine. The duration of surgery was around 140 minutes. The estimated blood loss was 350 ml and no blood transfusion was required. The patient remained hemodynamically stable and comfortable throughout the surgery (Figure 1B).

Postoperatively, pain management was done with 8 ml of 0.125% of epidural bupivacaine every 12 hourly and intravenous paracetamol 1 gram 8 hourly for 48 hours. The patient resumed oral feeding about six hours postoperatively. The epidural catheter was removed on the third postoperative day. The patient was discharged on the sixth postoperative day without any complications.

## Discussion

In this report, we have demonstrated that TEA is a reasonable and safe alternative to GA for MRM. Our patient had hypertension, reactive airway disease, and a difficult airway which precluded the safe conduct of GA. As such, we preferred TEA over GA as it involves blunting of stress response and avoidance of airway handling. Other potential advantages of TEA that have been widely reported in the literature include hemodynamic stability, lower analgesic consumption, superior postoperative analgesia, reduced postoperative nausea and vomiting (PONV), earlier resumption of feeding, and shorter duration of hospitalisation. Non-steroidal anti-inflammatory drugs (NSAIDs) have the propensity to precipitate acute asthmatic attacks. However, in our patient, adequate analgesia was achieved with epidural bupivacaine supplemented with intravenous paracetamol.

In general, the infrequent use of TEA for oncologic breast surgeries may be attributed to fear of potential complications like spinal cord injury, respiratory complications, spinal/epidural hematoma, and post-dural puncture headache. However, with experience and due precautions, these complications are extremely rare. The breast, axillary nodes, and pectoral muscles derive their innervation from multiple sources, including branches from the first to the sixth intercostal nerves, brachial plexus, intercostal brachial nerve (T2-T3), supraclavicular nerve, and lateral and medial pectoral nerves from the cervical plexus [3]. The supplementation of TEA with interscalene block in patients undergoing MRM has been shown to improve pain scores and reduce opioid consumption during the first 24 hours postoperatively [4]. Local anesthetic infiltration has been used along with TEA for axillary lymph node dissection [5]. The paravertebral block is another method that can be employed as an alternative to GA for mastectomies. Surgical anesthesia by thoracic paravertebral block alone, however, causes considerable patient discomfort during neural block and surgery. Ono et al. concluded that the combined use of paravertebral block and GA can be a preferable choice of anesthesia for patients undergoing major breast cancer surgery [6]. Cervical epidural anesthesia has also been used successfully for MRM, however, it is not recommended in patients with compromised pulmonary functions and poor cardiovascular reserve. Moreover, there is a possibility of paralysis of the phrenic nerve with a cervical epidural block [7].

Adequate epidural analgesia in the postoperative period has been shown to improve the perioperative outcome in patients at high risk for postoperative pulmonary complications [8]. Ropivacaine has a better safety profile than bupivacaine and may be a better choice during TEA. However, at lower doses, bupivacaine is equally safe. Previous reports have shown a high incidence of pruritus and urinary retention with TEA. However, all such reports involved the addition of opioids as an adjuvant with a local anesthetic. Epidural fentanyl produces more segmental analgesia with a reduced risk for delayed respiratory depression, unlike hydrophilic morphine which also produces late respiratory depression necessitating intensive monitoring in the postoperative period [9]. In our case, we avoided the addition of an opioid with bupivacaine. Our case reinforces the previous reports of MRM which were successfully conducted under TEA (Table 1).

**Table 1:** Summary of previous reports of oncologic breast surgeries conducted under thoracic epidural anesthesia. TEA: thoracic epidural anaesthesia; GA: general anaesthesia; PONV: postoperative nausea and vomiting; TRAM: transrectus abdominal muscle; CA: cancer

Clinical Research	Procedure for TEA	Results
TEA (n=60) versus GA (n=72) [8].	Initial dose of 8 to 14 ml of 2% lidocaine with 1:200,000 epinephrine [without TRAM] and 20 ml [with TRAM] followed by maintenance of anesthesia with 2-5 ml of 2% lidocaine with 1:200,000 epinephrine every 20 to 40 minutes through the epidural catheter.	TEA group had earlier hospital discharge and lesser incidence of PONV as compared to GA group. One patient in TEA had transient apnea, unresponsiveness and bradycardia (32 beats/ min) after administration of intravenous fentanyl (100 µg) and midazolam (2mg) and 12 ml of 2% lidocaine with 1:200, 000 adrenaline through epidural catheter.
TEA (n=30) versus GA (n= 30) [10].	Initial dose of 5–10 ml of 0.2% ropivacaine. Subsequent 3–5-ml doses of ropivacaine, if sensory or motor block (Bromage scale) was not adequate. Postoperative pain in the TEA group was controlled with the continuous infusion of 4–6 ml/hour of 0.2% ropivacaine through the epidural catheter for the first 24 hours. For the second 24 hour, the epidural infusion was reduced to 3–4 ml/hour.	Lower incidence of PONV, rapid post-anesthesia recovery, better postoperative analgesia and higher patient satisfaction scores were observed in TEA group as compared to GA group.
TEA + ipsilateral interscalene block (n=25) versus GA (n= 25) [11].	10-15 ml of 0.2% ropivacaine was injected, then interscalene block was done with 8 ml of 0.2% ropivacaine. Anesthesia was maintained with 5-10 ml of 0.2% ropivacaine per hour	The verbal rating scale and analgesic requirement were significantly lower in the TEA group as compared to GA group. Patients' satisfaction was greater with TEA than with GA.
TEA (n=20) versus GA (n=20) [12].		TEA was successful in all 20 patients. Lower incidence of PONV, superior quality of postoperative analgesia, lower analgesic consumption and lesser duration of hospital stay were seen in TEA group versus GA group. Higher incidence of pruritus (55%) was noted in TEA group versus GA group (0%).
TEA (n= 450) [13]	TEA was initiated with a bolus of 0.375% or 0.5% ropivacaine 15 ml injected slowly. Intraoperatively, additional doses of 0.375% or 0.5% ropivacaine 5-6 ml were given through the catheter at two-hour intervals.	TEA was successful in 439 patients. Six patients (1.4%) developed Horner's syndrome. All signs of Horner's syndrome resolved gradually within 180 mins of discontinuing the epidural infusion.
Case Report		
56-year-old female/CA of the right breast/advanced cryptogenic fibrosing alveolitis, ischemic heart disease, corticosteroid-induced hypertension, diabetes mellitus and difficult airway [9].	A loading dose of 7 ml of 0.5% bupivacaine followed by supplementation with supraclavicular nerve blockade by infiltrating subcutaneously 10 ml of 0.25% bupivacaine. Intraoperatively, the medial and lateral pectoral nerves beneath the pectoral muscles were blocked by the surgeon with 5 ml of 0.25% bupivacaine. Postoperatively, a total of five doses of morphine (2 mg/bolus) were administered through epidural catheter in the first 3 days.	Intraoperatively, hemodynamically stable TEA. She developed urinary retention on the first postoperative day which required catheterization. The epidural catheter was removed on the fourth postoperative day.
52-year-old female/CA of the right breast/hypertensive, diabetic, asthmatic [14].	A loading dose of 7 mL of 2% lidocaine with adrenaline 1:200,000 was given in 4 ml and 3 ml aliquots at 5-minute intervals followed by a continuous infusion of 2% lidocaine-adrenaline at 4 ml/hour	Intraoperatively, hemodynamically stable TEA. Postoperative analgesia was maintained with continuous infusion of 0.5% lidocaine-adrenaline at 4 ml/hour for 48 hours.
31-year-old female/CA of the left breast/history of left sided pneumonectomy/pulmonary arterial hypertension (40 mmHg) [15].	For TEA, single-shot 12 ml 0.5% injection ropivacaine at T6–T7 level. In addition, interscalene block was given on left side with 10 ml 0.5% ropivacaine to cover any axillary sparing.	Intraoperatively, hemodynamically stable TEA. Postoperative analgesia was maintained with 10 ml 0.2% ropivacaine 8 hours after surgery and 12 hourly thereafter for 36 hours, following which, the epidural catheter was removed.

## Conclusions

Thoracic epidural anesthesia is a feasible and safe alternative to general anesthesia for oncologic breast surgeries particularly in high-risk patients having co-morbidities and difficult airways. The use of thoracic epidural anesthesia for breast surgery could enhance postoperative outcomes and curtail the overall cost of these procedures. Based on the available evidence, we recommend thoracic epidural anesthesia for major breast surgeries in high-risk patients, though, large, randomised control trials are the need of the hour.
